# Comparative Analysis of the Orphan CRISPR2 Locus in 242 *Enterococcus faecalis* Strains

**DOI:** 10.1371/journal.pone.0138890

**Published:** 2015-09-23

**Authors:** Karthik Hullahalli, Marinelle Rodrigues, Brendan D. Schmidt, Xiang Li, Pooja Bhardwaj, Kelli L. Palmer

**Affiliations:** Department of Biological Sciences, The University of Texas at Dallas, Richardson, Texas, United States of America; University of Kansas, UNITED STATES

## Abstract

Clustered, Regularly Interspaced Short Palindromic Repeats and their associated Cas proteins (CRISPR-Cas) provide prokaryotes with a mechanism for defense against mobile genetic elements (MGEs). A CRISPR locus is a molecular memory of MGE encounters. It contains an array of short sequences, called spacers, that generally have sequence identity to MGEs. Three different CRISPR loci have been identified among strains of the opportunistic pathogen *Enterococcus faecalis*. CRISPR1 and CRISPR3 are associated with the *cas* genes necessary for blocking MGEs, but these loci are present in only a subset of *E*. *faecalis* strains. The orphan CRISPR2 lacks *cas* genes and is ubiquitous in *E*. *faecalis*, although its spacer content varies from strain to strain. Because CRISPR2 is a variable locus occurring in all *E*. *faecalis*, comparative analysis of CRISPR2 sequences may provide information about the clonality of *E*. *faecalis* strains. We examined CRISPR2 sequences from 228 *E*. *faecalis* genomes in relationship to subspecies phylogenetic lineages (sequence types; STs) determined by multilocus sequence typing (MLST), and to a genome phylogeny generated for a representative 71 genomes. We found that specific CRISPR2 sequences are associated with specific STs and with specific branches on the genome tree. To explore possible applications of CRISPR2 analysis, we evaluated 14 *E*. *faecalis* bloodstream isolates using CRISPR2 analysis and MLST. CRISPR2 analysis identified two groups of clonal strains among the 14 isolates, an assessment that was confirmed by MLST. CRISPR2 analysis was also used to accurately predict the ST of a subset of isolates. We conclude that CRISPR2 analysis, while not a replacement for MLST, is an inexpensive method to assess clonality among *E*. *faecalis* isolates, and can be used in conjunction with MLST to identify recombination events occurring between STs.

## Introduction

*Enterococcus faecalis* is a gram-positive lactic acid bacterium and opportunistic pathogen that normally colonizes the intestinal tracts of humans and other hosts [1]. It is used in the production of fermented foods and live probiotics, and as an indicator of fecal contamination in beaches and recreational waters [2]. *E*. *faecalis* has gained notoriety for causing healthcare-associated infections (HAIs) such as catheter-associated bloodstream infections, urinary tract infections, and surgical site infections [3]. The horizontal acquisition of antibiotic resistance genes by some *E*. *faecalis* strains has reduced treatment options for these infections [4,5].

Many studies seek to determine whether *E*. *faecalis* strains causing infection outbreaks are clonal. This information is important for infection control. Pulsed-field gel electrophoresis (PFGE), which assesses banding patterns resulting from restriction enzyme digestion of genomic DNA, has been widely employed to assess clonality among *E*. *faecalis* strains. However, poor reproducibility between labs is a drawback to the technique. Multi-locus sequence typing (MLST) is a commonly used method of assessing subspecies phylogenetic relationships. In one *E*. *faecalis* MLST scheme [6], internal regions of seven housekeeping genes (*gdh*, *yqiL*, *aroE*, *pstS*, *gyd*, *xpt*, *gki*) are amplified by PCR, sequenced, and compared to a global database (http://pubmlst.org/efaecalis/) such that an allele variant number can be assigned to each gene. Based on the combination of allele numbers, a sequence type (ST) is assigned to a strain. Analysis of whole genome sequences has also been used to assess phylogenetic relationships among *E*. *faecalis* strains [7–11], although the number of strains queried to date is much lower than that assessed by MLST. While whole genome sequencing is increasingly cost-effective and attractive for population studies of infection outbreaks, the technology and expertise required to generate and analyze data currently limits this approach to facilities with appropriate resources for such studies.

**Fig 1:**
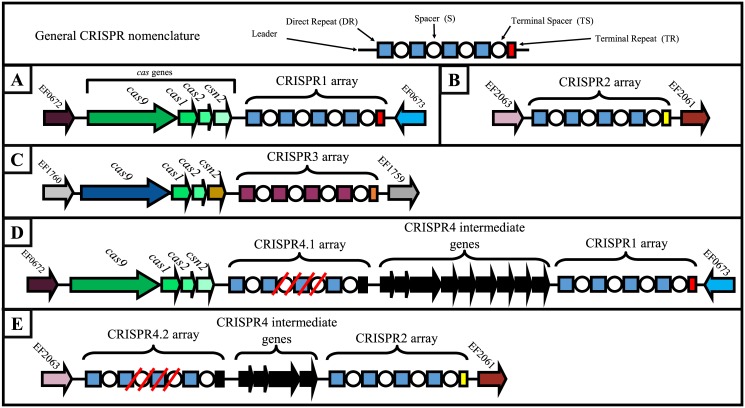
*E*. *faecalis* CRISPR locus architecture. All known CRISPR loci in *E*. *faecalis* are shown: A) CRISPR1-*cas*, B) CRISPR2, C) CRISPR3-*cas*, D) CRISPR4.1, and E) CRISPR4.2. Repeats are indicated by squares and spacers are indicated by circles; the array size shown is intentionally arbitrary. The terminal degenerate repeat is indicated a rectangle. Identical colors are indicative of identical or closely related sequences. Genes and terminal repeats that are associated only with CRISPR4 are shown in solid black. CRISPR4 occurs as two variants: a CRISPR1-*cas*-associated variant and a CRISPR2-associated variant, denoted as CRISPR4.1 and CRISPR4.2 respectively. Red cross-hatches indicate gaps in the genome assembly in the CRISPR4. CRISPR4 loci were present in 3 *E*. *faecalis* genomes examined; B-4-111 possesses the CRISPR1 variant while ATCC 6055 and B16457 possess the CRISPR2 variant. Locus identifiers for CRISPR4 intermediate genes in B-4-111 are WOA_00879-WOA_00888. Locus identifiers for CRISPR4 intermediate genes in ATCC 6055 are WOU_01828-WOU_01823.

CRISPR (Clustered, Regularly Interspaced, Short Palindromic Repeats) typing systems have been developed for several pathogens (reviewed recently by Shariat and Dudley, [12]). CRISPR analysis can be used alone or in conjunction with MLST to investigate subspecies phylogenetic relationships. CRISPR typing relies on sequencing or probing of CRISPR arrays present in microbial genomes. CRISPR arrays consist of short identical repeats separated by sequences called spacers (Fig 1). The number of spacers contained within CRISPR arrays can vary widely (from 1 to >100, depending on the species studied). CRISPR arrays are components of CRISPR-Cas systems, which function as adaptive immune systems in prokaryotes [13]. In brief, when challenged with a mobile genetic element (MGE) such as a plasmid or phage, a cell may incorporate a short segment (protospacer) of the MGE genome into the CRISPR array as a novel spacer. Spacer addition occurs at one end of the CRISPR (the 'leader' end), thereby establishing a temporal record of spacer acquisitions, with the terminal spacer being the oldest in the array. The adaptation event requires the CRISPR-associated (Cas) proteins Cas1 and Cas2 and a short (2 to 5 nucleotide) sequence called a protospacer adjacent motif (PAM). Since selection of the spacer sequence is dependent only on the PAM and the presence of replication forks (this second shown in a Type I CRISPR-Cas system) [14], the abundance of each on plasmids and phage ensure that identical spacers will be infrequently acquired by different cells, meaning that the convergent evolution of CRISPR2 arrays is unlikely. Small RNAs derived from transcription of CRISPR arrays direct Cas nucleases to interfere with MGEs of similar sequence. Overall, the CRISPR array serves as a molecular memory of past MGE encounters. Spacers are added and deleted, arrays can rarely be exchanged by recombination, and spacers can accumulate polymorphisms over time [12,15]. CRISPR typing systems use shared spacers (memories) and spacer order across strains to quantify relatedness [12].

CRISPR-Cas systems are categorized into three major types (I, II, and III), with each having a type-specific *cas* gene [16]. The CRISPR-Cas systems identified in *E*. *faecalis* genomes to date are Type II systems, which possess the type-specific gene *cas9*. Two Type II CRISPR-Cas systems, designated CRISPR1-Cas and CRISPR3-Cas, have been identified for *E*. *faecalis* [11,17] (Fig 1). These systems possess 36 bp repeat sequences interspersed by 30 bp spacer sequences, with the terminal repeat in the array being degenerate in sequence. The distributions of CRISPR1-Cas and CRISPR3-Cas vary across the *faecalis* species, with the systems being absent from most multidrug-resistant strains [17]. A third CRISPR locus, referred to as CRISPR2, is an orphan locus devoid of *cas* genes but of variable spacer composition that has been identified in every *E*. *faecalis* genome studied to date ([17] and this study). The direct repeat sequences of CRISPR1 and CRISPR2 are identical, suggesting that the CRISPR1-Cas and CRISPR2 loci are functionally linked and/or of shared ancestry [11,17]. Enterococcal CRISPR-Cas systems, including CRISPR1-Cas, can be gained and lost by recombination of chromosomal DNA via a Hfr-like mechanism [7,17,18], providing a potential mechanism for occasional spacer addition to CRISPR2. The preservation of the orphan CRISPR2 locus in *E*. *faecalis* as well as its diversity in terms of spacer content makes it an attractive target for phylogenetic studies of *E*. *faecalis* isolates.

Here, we perform a comparative analysis of CRISPR2 sequences from 228 *E*. *faecalis* genomes. We investigate the relationship between CRISPR2 sequence, ST assigned by MLST, and genome phylogeny. We additionally perform MLST and CRISPR2 analysis on 14 previously untyped *E*. *faecalis* central line-associated bloodstream infection (CLABSI) isolates. We find that, while CRISPR2 analysis does not possess the same discriminatory power as MLST for all *E*. *faecalis* isolates, clonality can be assessed and certain STs can be accurately predicted by amplification and sequencing of the CRISPR2 locus. The significance of this is that CRISPR2 analysis can yield useful data on the clonality of *E*. *faecalis* isolates when funds for such studies are limited (i.e., sequencing of one allele versus the seven required for MLST). Moreover, when MLST data are available, the addition of the CRISPR2 allele can yield useful information about recombination events between STs.

## Materials and Methods

### Bacterial strains and genome sequences

Bacterial strains and genome sequences used in this study are shown in the S1 Dataset. For *in silico* CRISPR analysis, complete and draft *E*. *faecalis* genomes were downloaded from GenBank in 2013. *E*. *faecalis* genomes with sequence gaps in the CRISPR2 array were omitted from further study (with the exception of strain R712) and are not shown in the S1 Dataset. *E*. *faecalis* HIP11704 [19], was used as a positive control strain for CRISPR2 amplification studies. A collection of 14 previously untyped *E*. *faecalis* bloodstream isolates was also examined [20]. Strains were routinely cultured on brain heart infusion (BHI) agar or broth medium at 37°C.

### CRISPR identification

The three previously identified CRISPR loci in *E*. *faecalis* [11,17] occur at conserved positions in the *E*. *faecalis* genome, between orthologs of the V583 ORFs EF0672 and EF0673 (for CRISPR1-*cas*), EF2063 and EF2061 (for CRISPR2), and EF1760 and EF1759 (for CRISPR3-*cas*) (Fig 1). BLAST analysis was used to identify these CRISPR-flanking genes in *E*. *faecalis* genomes. CRISPR repeats and spacers were manually annotated. The consensus repeat sequences for CRISPR1, CRISPR2, and CRISPR3 were previously published [17]. A new CRISPR locus, which we have named CRISPR4, was identified in 3 strains (B-4-111, ATCC 6055, and B16457). CRISPR4 was identified based on its proximity to CRISPR1 or CRISPR2 (Fig 1).

### Analysis of CRISPR2 spacers

CRISPRtionary [21] was used to generate a dictionary of CRISPR2 spacers and to assign each unique spacer a number. The CRISPRtionary generated for our 228 CRISPR2 sequences is shown in the S2 Dataset. Clustal Omega was used to examine spacer similarity and identify single nucleotide variants (SNVs) [22,23].

### *In silico* MLST analysis

Where available, MLST data were gathered from the literature (see the S1 Dataset for references and a summary of information available for each strain). STs for the remaining genomes were manually identified by *in silico* MLST (http://pubmlst.org/efaecalis/). Ten genomes could not be assigned an ST because they possess novel alleles not represented in the MLST database. Their ST is shown as unknown. BURST analysis was performed through the MLST database.

### Statistical analyses of diversity

Calculation of Simpsons indices, adjusted Rand coefficients, and adjusted Wallace coefficients were performed as previously described [24,25] using an online tool (http://darwin.phyloviz.net/ComparingPartitions/index.php?link=Tool). The input data are described in the results section.

### Construction of genome sequence-based phylogenetic tree

**Fig 2:**
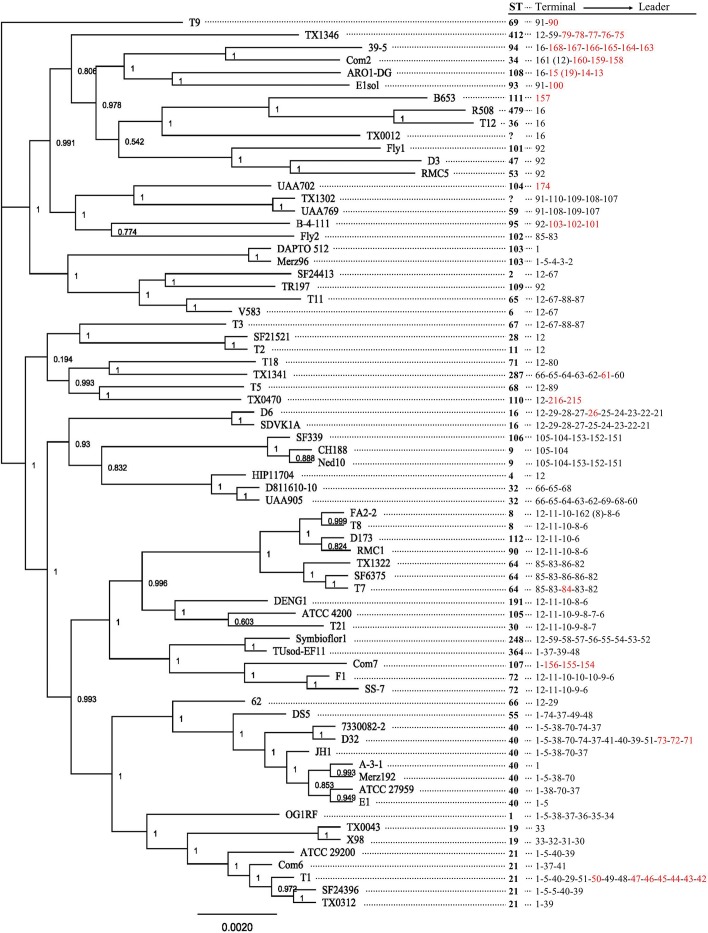
Genome sequence-based phylogeny. The FastTree output is shown with local bootstrap support on respective nodes. CRISPR2 array type and ST are indicated. Unknown STs are shown with “?”. CRISPR2 arrays are organized from terminal spacer to leader spacer. Spacers in red are strain-specific. Spacers that have numbers in parentheses next to them indicate Single Nucleotide Variants (SNVs). For example, a spacer labeled 161 (12) indicates that spacer 161 is a SNV of spacer 12.

For whole genome phylogenetic analysis, we utilized genome sequences of 71 representative strains (Fig 2). The ProgressiveMauve algorithm was used to align each genome [26]. In total, 2975 local collinear blocks (LCBs) were identified. To extract sequence for a genome sequence-based phylogeny, we only considered LCBs that were present in all 71 strains for further analysis. These 183 LCBs were further curated by only including those for which ≥90% of the columns of the alignment were identical or for which the sequence length was >30,000 bp. For the latter criterion, we only included LCBs for which the minimum sequence length was ≥80% of the maximum sequence length. The remaining 15 LCBs (~660 kbp sequence in total) were concatenated and used as the input alignment for FastTree [27] with the GTR (General Time Reversible) model and default 1000 replicates.

### CRISPR2 and MLST assessment of 14 *E*. *faecalis* bloodstream isolates

The 14 *E*. *faecalis* bloodstream isolates were described in a previous study [20]. The isolates were confirmed to be *E*. *faecalis* using *ddl* PCR as previously described [28,29]. Genomic DNA was purified from *E*. *faecalis* using the Ultraclean Microbial DNA Isolation Kit (MoBio). The entire CRISPR2 region was amplified to determine CRISPR2 size and for DNA sequencing, using the primers CR2 For (5’-ACTTATCACTTGATTAGTTTTCG-3’) and CR2 Rev, (5’-GTGATGCGAATACGGAATCATGG-3’) with Taq polymerase (New England Biolabs) and 55° annealing temperature. Previously published *E*. *faecalis*-specific *ddl* primers were used for positive control reactions with 50° annealing temperature [28,29]. MLST was performed as described previously [6], except that two alternative *gdh* primers were used for DNA sequencing purposes (gdh for, 5'-TTCTATGGAGCCTCCTGTTGC-3' and gdh rev, 5'-AAGTTTGTACTATCGCCATTTAGGC-3'). DNA sequencing was performed at the Massachusetts General Hospital DNA Facility.

## Results and Discussion

### *E*. *faecalis* genomes used in this study

The 228 *E*. *faecalis* strains in our collection represent 62 STs, and their isolation dates range from the early 20^th^ century to present (S1 Dataset). Of the 228 strains, 82 are ST6, which is the most commonly occurring ST in our collection. For 10 of the 228 strains, ST could not be assigned due to novel alleles being present. For all strains, information compiled from the literature and genome sequence databases is shown in the S1 Dataset. Based on limited availability of information for certain strains, it is possible that our dataset contains serial clinical isolates (i.e., isolates from the same patient over time), particularly for the large number of ST2 and ST6 strains in our collection. That said, four known serial isolates from a single patient are present in our collection (DAPTO 512, DAPTO 516, S613, and R712; [30,31]), and each of these strains has a similar but not identical CRISPR2 (discussed further below). Therefore serial isolates also provide valuable information on CRISPR2 structure.

### Generation of a CRISPR2 spacer dictionary

CRISPR2 loci were not automatically annotated in the genome sequences of our 228 strains. Therefore we manually annotated them, using BLAST analysis to identify the genes that flank the conserved location of CRISPR2 in *E*. *faecalis* genomes (Fig 1). All annotated CRISPR2 were then analyzed with CRISPRtionary [21], a tool that generates a dictionary of unique spacer sequences present in CRISPR arrays. The raw CRISPRtionary output is shown in the S2 Dataset. CRISPRtionary assigns an arbitrary number to each unique spacer. These number assignments are useful in that we can determine whether specific numbers (and therefore specific CRISPR spacers) are unique to or diagnostic for a particular lineage.

Among the 228 strains, the number of spacers in CRISPR2 ranges from 1 to 14. In total, 710 CRISPR2 spacers were identified. Removing redundancies (i.e., the same spacer present in multiple strains), 148 unique spacer sequences occurred. The CRISPRtionary number assignments for all 148 unique CRISPR2 spacer sequences are shown in the S2 Dataset. Among those, 63 were strain-specific, being present in only 1 of the 228 strains. Generally, the most frequently occurring spacers in the collection were terminal spacers. This was anticipated, given that these are expected to be the oldest ‘memory’ in the CRISPR, and that terminal CRISPR spacers are observed to be highly conserved in other bacterial species [32–34].

The S3 Dataset shows the distribution of CRISPR2 spacers across our strain collection. For this analysis, strains with identical ST and identical CRISPR2 were consolidated under a representative strain, such that only unique combinations of ST and CRISPR2 sequence are shown. In describing the CRISPR2 loci, we refer to them from terminal spacer (TS) to leader spacer (i.e., from oldest to newest spacers). For example, all ST6 and ST2 strains in our collection possess a CRISPR2 containing two spacers which were assigned the arbitrary identifiers of 12 and 67 by CRISPRtionary (S3 Dataset). For this CRISPR2, spacer 12 is the terminal spacer (TS), and spacer 67 is the leader-proximal spacer. Therefore we refer to this CRISPR2 as TS12-S67 or simply 12–67.

### Occurrence of CRISPR1-Cas and CRISPR3-Cas

CRISPR1-*cas* occurs in 27 of 228 isolates, and CRISPR3-*cas* occurs in 15 of 228 isolates (S1 and S3 Datasets). CRISPR1-*cas* and CRISPR3-*cas* did not co-occur in any of the strains. Because most of the CRISPR1 and CRISPR3 loci contained sequence gaps, and we did not have the strains for independent sequencing and gap closure, CRISPR1 and CRISPR3 loci were not analyzed further. As anticipated [17], all strains from the ST2, ST6 and ST9 lineages, described as being high-risk [35], lacked CRISPR1-*cas* and CRISPR3-*cas*. Conversely, particular STs commonly possessed CRISPR1-*cas* and CRISPR3-*cas*: ST40 and ST21 for CRISPR1-*cas* and ST65 for CRISPR3-*cas*.

### Identification of a novel CRISPR locus, CRISPR4

We identified a novel *E*. *faecalis* CRISPR locus, which we have named CRISPR4 (Fig 1). CRISPR4 occurs in 3 of the 228 strains in our collection (B-4-111, ATCC 6055, and B16457). CRISPR4 is linked with either CRISPR1-*cas* or CRISPR2, as the CRISPR4 direct repeat sequence is identical to those of CRISPR1-*cas* and CRISPR2. However, a set of genes that lack identity to known *cas* genes is associated with CRISPR4, and the terminal repeat sequence associated with CRISPR4 is distinct from that of CRISPR1 and CRISPR2. We refer to the CRISPR1-associated CRISPR4 (occurring in strain B-4-111) as CRISPR 4.1. We refer to the CRISPR2-associated CRISPR4 (occurring in strains ATCC 6055 and B16457) as CRISPR 4.2. It is unlikely that CRISPR4 is an artifact of poor sequence assembly, based on the presence of the novel CRISPR-associated genes as well as that the terminal repeat sequence in the CRISPR4 array is unique to CRISPR4-containing strains. Additionally, CRISPR4 spacers were unique, i.e., they were not present in our CRISPR2 spacer dictionary.

### Observations on CRISPR2 and its relationship with MLST and a genome sequence-based phylogeny

The S3 Dataset shows ST and CRISPR2 spacer arrays for our collection of 228 strains, and Fig 2 shows CRISPR2 spacer arrays relative to a genome sequence-based phylogenetic tree generated for a representative 71 strains. Several direct and easily noticeable correlations between CRISPR2 sequence and ST can be made. For example, a correlation between ST and CRISPR2 is seen with ST2/ST6, for which all 101 isolates possess an identical CRISPR2 locus that is shared with only two isolates outside those STs (S3 Dataset). Another correlation between ST and CRISPR2 is seen for ST9. For the 10 ST9 isolates, all possess the CRISPR2 terminal spacer 105, which is present as a terminal spacer in only one other isolate in our collection, SF339, a ST106 isolate (S3 Dataset). The genome tree shows that SF339 and two representative ST9 isolates are of common ancestry (Fig 2), therefore TS105 may be a diagnostic sequence for this branch.

For other lineages, CRISPR2 is less discriminatory. Some CRISPR2 loci are not associated with specific STs; examples are small CRISPR2 loci possessing only a single spacer, such as those possessing only TS91 or TS16.

CRISPR2 spacer deletion and rearrangement were detected in the four known serial isolates in the genome collection (S3 Dataset). The four strains (DAPTO 512, DAPTO 516, S613, and R712) are the same ST (ST103) and were isolated consecutively over a period of 2 months from the same patient in 2004 in Indiana [30]. Initially, DAPTO 512 and DAPTO 516 were isolated in May 2004 prior to antibiotic treatment and were ampicillin-susceptible and vancomycin-resistant. Subsequently, linezolid followed by oral ciprofloxacin were administered, and upon hospitalization of the patient due to fever a few days later, S613 was isolated. Treatment with daptomycin and amikacin was initiated. Two weeks later, the patent was rehospitalized for a fever and R712 was isolated from blood culture. Merz96, was isolated two years prior in Maryland [36]. The CRISPR2 of Merz96 is TS1-S5-S4-S3-S2, and the four serial isolates from Indiana each possess a different variation of this locus (S3 Dataset). The variations appear to be deletions or rearrangements of the Merz96 CRISPR2 array. ST103 strains with varying CRISPR2 loci may have co-existed in the patient. Another possibility is that only the CRISPR2 array of Merz96 pre-existed in the patient, and then diversification of CRISPR2 by rearrangements and deletions occurred as a result of DNA damage from antibiotic-induced stress. An alternative and technical explanation is that the CRISPR2 arrays of these strains were not assembled correctly during genome sequencing; however, because 100 base reads were used for assembly [31], this is unlikely.

In examining CRISPR2 distribution across the genome tree (Fig 2), evidence for the horizontal transfer of CRISPR2 arrays is present. For example, ST64 and ST102 possess similar CRISPR2 arrays with TS85 and S83, but the ST64 and ST102 representatives are not co-localized on our tree. Other examples are ST19 and ST66, which occur within the large clade containing ST40 and ST21 but possess CRISPR2 loci of entirely different spacer compositions than ST40 and ST21. Based on our current understanding of the population structure of *E*. *faecalis* [6,37], recombinatorial exchange of CRISPR2 arrays is expected. The conservation of CRISPR2 in certain lineages, such as ST2/ST6, may indicate that these strains are primarily exchanging DNA with each other due to their prevalence in hospitals, although this is highly speculative.

### Quantification of the discriminatory power and diagnostic utility of CRISPR2

For the purposes of performing statistical analyses to compare the resolution of MLST and CRISPR2 analysis, here we define a CRISPR2 type. We identified three steps to determine the type of a CRISPR2 array. The rationale for this is based on observations from other large-scale genomic analyses of Type II CRISPR-Cas systems. Because terminal spacers, as the oldest ‘memory’ in the array, tend to be highly conserved [34,38–40], we first clustered CRISPR2 arrays by shared terminal spacers. A second level of clustering was then performed based on the spacer immediately adjacent to the terminal spacer (for CRISPR2 arrays with more than spacer). Finally, because internal spacer deletions are frequently observed [34,38–40], thereby preserving the oldest and newest memories, we finally added any remaining CRISPR2 arrays to existing clusters based on shared, non-terminal, spacers. Note that this third criterion is very liberal in defining CRISPR2 types; as such, some discriminatory power of CRISPR is likely lost. The S3 Dataset shows CRISPR2 types assigned to each CRISPR2 array in our genome analysis. Each array is named by the terminal spacer ID, then a letter is assigned based on the subsequent clustering performed. It is noted in the S3 Dataset where CRISPR2 array structure may be diagnostic for certain STs or lineages. This was observed within some CRISPR2 types.

The original MLST study on *E*. *faecalis* in 2006 defined groups of STs that differed by no more than two alleles as Clonal Complexes (CCs). For example, ST2 and ST6 are double locus variants (DLVs), and these STs along with all of their single locus variants (SLVs) were categorized as CC2, with the defining number (in this case, 2) being the ST with the greatest number of SLVs (i.e., the predicted founder). However, many more STs have been deposited since the original study, so instead of using the previous nomenclature regarding CCs, we used the BURST algorithm provided by the MLST database to classify STs into groups. Groups were defined as all STs that shared *n* alleles. When *n* = 6, the predicted group founders were identical to the CCs previously identified for the STs in our collection [6]. For STs that lacked a group (singletons), the *n* = 6 founder was defined as the ST. For STs that had a group but lacked a group founder, the ST with the most SLVs was chosen as the founder. If there was no ST with the most SLVs, a random ST was chosen as the group founder. The end result was a system that offered an identical degree of discrimination and classification as the previously defined clonal complexes. An *n* = 5 group was also included in our analyses for comparative purposes.

We used the Simpsons Index of Diversity (SID) [41] to determine the level of discrimination offered by each of our typing methods. SID values indicate the probability of two random isolates belonging to different types. SID values for ST, the *n* = 6 group, and the *n* = 5 group were 0.845, 0.776, and 0.654, respectively. The SID value for our CRISPR typing definition was 0.735, indicating that CRISPR2 has similar discriminatory ability as the n = 6 group. Furthermore, the Adjusted Rand coefficient (AR) was used as previously described [24] to assess the level of congruence between CRISPR2 type and MLST. AR values for ST, the *n* = 6 group, and the *n* = 5 group in relation to CRISPR2 were 0.673, 0.877, and 0.702, respectively, further supporting a strong relationship between CRISPR2 type and the *n* = 6 group. Finally, the Adjusted Wallace coefficient (AW) was calculated to further assess these relationships [25]. The AW_CRISPR2_→_ST_ = 0.507 and AW_CRISPR2_→_n = 6_ = 0.790, indicating that if two strains are related by CRISPR2 type, there is a 51% chance they belong to the same ST and a 79% chance they belong to the same *n* = 6 group. Conversely, AW_ST_→_CRISPR2_ = 0.999 and AW_n = 6_ →_CRISPR2_ = 0.986, indicating that if two strains belong to the same ST or *n = 6* group, there is nearly a 100% chance they are identical in CRISPR2 type. Statistical analyses are shown in tabular form in the S3 Dataset. Taken altogether, our data reveal that CRISPR2 is best suited to predict the *n* = 6 group, even though the latter is more discriminatory. Because high-risk lineages are often defined at the level of clonal complexes, thereby making the *n* = 6 group useful in epidemiological studies, CRISPR2 analysis may be a useful assessment to identify these lineages.

Even though ST is much more discriminatory than CRISPR2, the orphan locus still has distinctive advantages. CRISPR2 relates strains that are difficult to relate or unrelatable by ST. Specifically, ST1, ST40, ST21, and ST55 all possess identical CRISPR2 types and are closely related by our genome tree. However, none of these STs belong to the same *n* = 6 group and only ST55 and ST40 belong to the same *n* = 5 group.

### CRISPR2 and MLST analysis of 14 *E*. *faecalis* CLABSI isolates

We used CRISPR2 analysis along with MLST to assess clonality among a collection of previously untyped *E*. *faecalis* bloodstream isolates [20]. These strains were recovered from CLABSI patients at the Johns Hopkins Medical Institute between January 2012 and September 2013 [20].

We amplified and sequenced the CRISPR2 locus of each strain, and used those data to assess clonality of the isolates. Six different CRISPR2 amplicon sizes were observed for the 14 isolates (data not shown). Sequencing of the products and comparison with our CRISPRtionary spacer bank (shown in the S2 Dataset) allowed us to assign spacer numbers to each of the CRISPR arrays (Table 1). Note that all spacers were already represented in our CRISPRtionary spacer bank (i.e., no novel spacers were identified). From the CRISPR2 sequence data, we concluded that 4 isolates of CRISPR2 array structure 85-83-86-82 were clonal, as were 2 isolates having CRISPR2 array structures of 12–21 and 12-29-28-27-26-25-21.

**Table 1:** MLST and CRISPR2 analysis of 14 *E*. *faecalis* bloodstream isolates.

Isolate	CRISPR2 Type	Predicted ST	Actual ST
**ST accurately predicted using CRISPR2 sequence alone**			
**2763**	33-32-31-30	19	19
**3729**	1-5-38-70	40	40
**4269**	12-29-28-27-26-25-21	16	16
**5900**	12–21	16	16
**6710**	1-5-4-3-2	103	103
**ST not accurately predicted using CRISPR2 sequence alone**			
**660**	85-83-86-82	64	179
**5606**	85-83-86-82	64	179
**6358**	85-83-86-82	64	179
**6830**	85-83-86-82	64	179
**ST could not be predicted using CRISPR2 sequence alone**			
**1999**	16	-	669
**2867**	16	-	257
**4431**	12-9-8	-	79
**6709**	92	-	109
**7047**	91	-	261

To compare CRISPR2 analysis with MLST for these isolates, we compared the CRISPR2 arrays of the 14 isolates with those in Fig 2 and the S3 Dataset, and predicted STs where possible for the 14 isolates (Table 1). We then performed MLST on the 14 isolates (Table 1). Our assessments of clonality using CRISPR sequence alone, described above, were supported by MLST (Table 1). Specifically, strains having CRISPR2 type 85-83-86-82 were all the same ST (ST179), and strains having CRISPR2 types of 12–21 and 12-29-28-27-26-25-21 were the same ST (ST16). Although ST was only accurately predicted for 5/14 isolates, clonality was completely identified, suggesting that CRISPR2, although not a replacement for MLST, is sufficient to rapidly assess clonality at this scale.

We next compared our ST predictions made from CRISPR2 analysis alone. For the 9 isolates for which we predicted ST using CRISPR2 analysis, 5 were found to be accurate by MLST (Table 1). The 4 incorrectly predicted STs possessed the array TS85-S83-S86-S82 and were all ST179, rather than ST64 as predicted by our CRISPR2 analysis. Note that ST179 was not represented in our 228 genome analysis (S3 Dataset). This discrepancy between prediction and observation can be explained by examining the MLST alleles further (S1 Table). ST179 is a single MLST locus (*xpt*) and CRISPR2 variant of ST16. The *xpt* gene (EF2365) is the closest MLST locus downstream of CRISPR2, and ST179 and ST64 share common *xpt* and CRISPR2 alleles. Recombination between ST16 and ST64 may have exchanged the CRISPR2 and *xpt* loci to create ST179, which possesses the ST64 *xpt* and ST64 CRISPR2, but is identical to ST16 at other MLST loci. This result demonstrates that CRISPR2 analysis adds additional phylogenetic signal to *E*. *faecalis* MLST and can provide evidence for horizontal transfer events between what appear to be distantly related lineages (such as ST64 and ST16). Taken altogether, CRISPR2 analysis on this level yielded as much information as MLST regarding the clonality of individual isolates, and when used in conjunction with MLST predicted recombination events between lineages.

## Conclusions

In this study we used *in silico* analysis of 228 genome sequences and laboratory investigation of a previously untyped collection of 14 CLABSI isolates to assess the utility of CRISPR2 analysis in phylogenetic studies of *E*. *faecalis*, which could be used alone or in conjunction with MLST. We undertook this analysis because CRISPR2 is a conserved feature of *E*. *faecalis* genomes that embodies the multiple allelic structure (where each spacer is an allele) of MLST while also being a single locus for the purposes of PCR amplification and DNA sequencing. We found that certain spacers or spacer configurations are diagnostic for several notable lineages among the 242 strains sampled in total here. Further, we found that amplification and sequencing of CRISPR2, followed by comparison with the CRISPRtionary sequences in the S2 Dataset and phylogenetic analyses in the S3 Dataset and Fig 2, yielded valuable information about the clonality and ST of previously untyped *E*. *faecalis* clinical isolates. Although CRISPR2 analysis is not a replacement for MLST, CRISPR2 analysis could be used for relatively inexpensive studies to assess clonality of clinical isolates, or to provide additional depth in phylogenetic analyses when MLST is performed.

An important caveat to this analysis is that CRISPR2 amplicon size alone cannot be used to assess clonality among a collection of novel isolates; sequencing of CRISPR2 products to determine CRISPR spacer sequences is required. That said, PCR reactions targeting the CRISPR2 loci of specific STs (such as that occurring in ST2/ST6) or a variety of blotting and hybridizing schemes such as the CRISPOL assay [33] could be used to examine spacer content. Further study will be required to develop and vet such approaches.

## Supporting Information

S1 Table MLST allele numbers and CRISPR2 structure for *E*. *faecalis* ST16, ST179, and ST64. (PDF)

S1 Dataset *E*. *faecalis* genomes analyzed in this study with strain metadata where available. (XLSX)

S2 Dataset (A) CRISPR2 arrays from each genome analyzed in this study. (B) CRISPR2 spacer dictionary. (XLSX)

S3 Dataset (A) CRISPR2 array structure and type assignment for 228 *E*. *faecalis* genomes. (B) Summary of statistical analyses comparing CRISPR2 typing with MLST. (XLSX)
