# Supplementary material for: Comparative Analysis of the Orphan CRISPR2 Locus in 242 Enterococcus faecalis Strains
Source: PLoS One. 2015 Sep 23;10(9):e0138890. doi: 10.1371/journal.pone.0138890 (PMC4580645; doi:10.1371/journal.pone.0138890)
Supplement: S1 Table — (PDF) [file pone.0138890.s001.pdf]

Table S1. MLST allele numbers and CRISPR2 type for *E. faecalis* ST16, ST179, and ST64.

		ST Allele Numbers		
**Locus Tag**	**Allele**	**ST16**	**ST179**	**ST64**
EF1004	*gdh*	5	5	10
EF1364	*yqiL*	6	6	4
EF1561	*aroE*	7	7	5
EF1705	*pstS*	1	1	11
EF1964	*gyd*	1	1	1
	CRISPR2	12-…-21	85-83-86-…	85-83-86-…
EF2365	*xpt*	7	1	1
EF2788	*gki*	3	3	16
